# Twenty-Four-Hour Urinary Aldosterone Predicts Inappropriate Left Ventricular Mass Index in Patients with Primary Aldosteronism

**DOI:** 10.1155/2013/294594

**Published:** 2013-12-29

**Authors:** Chi-Sheng Hung, Yi-Lwun Ho, Yi-Yao Chang, Vin-Cent Wu, Xue-Ming Wu, Jen-Kuang Lee, Shih-Chieh Chueh, Yen-Hung Lin, Yuan-Shian Changh, Shao-Yu Yang, Ya-Hui Hu, Ming-Jai Sui, Ming-Fong Chen, Kwan-Dun Wu

**Affiliations:** ^1^Telehealth Center, National Taiwan University Hospital, No. 7 Chung-Shan S. Road, Taipei 100, Taiwan; ^2^Department of Internal Medicine, National Taiwan University Hospital and National Taiwan University College of Medicine, No. 7 Chung-Shan S. Road, Taipei 100, Taiwan; ^3^Department of Internal Medicine, Taoyuan General Hospital, Taoyuan 330, Taiwan; ^4^Department of Laboratory Medicine, Far Eastern Memorial Hospital, New Taipei City 220, Taiwan; ^5^Department of Urology, National Taiwan University Hospital and National Taiwan University College of Medicine, Taipei 100, Taiwan; ^6^Department of Urology, Cleveland Clinic, Cleveland, OH 44011, USA; ^7^Department of Internal Medicine, Postal Hospital, Taipei 100, Taiwan; ^8^Internal Medicine, Buddhist Tzu Chi General Hospital, New Taipei City 231, Taiwan; ^9^Institute of Pharmacology, National Taiwan University Medical College, Taipei 100, Taiwan

## Abstract

*Objective*. Primary aldosteronism (PA) is associated with inappropriate left ventricular hypertrophy (LVH) in relation to a given gender and body size. There is no ideal parameter to predict the presence of LVH or inappropriate LVH in patients with PA. We investigate the performance of 24-hour urinary aldosterone level, plasma renin activity and aldosterone-to-renin ratio on this task. *Methods*. We performed echocardiography in 106 patients with PA and 31 subjects with essential hypertension (EH) in a tertiary teaching hospital. Plasma renin activity, aldosterone concentration, and 24-hour urinary aldosterone level were measured. *Results*. Only 24-hour urinary aldosterone was correlated with left ventricular mass index (LVMI) and excess LVMI among these parameters. The multivariate analysis revealed the urinary aldosterone level as an independent predictor for LVMI and excess LVMI. Analyzing the ability of urinary aldosterone, plasma aldosterone concentration, and plasma aldosterone-to-renin ratio to identify the presence of LVH (ROC AUC = 0.701, 0.568, 0.656, resp.) and the presence of inappropriate LV mass index (defined as measured LVMI in predicting LVMI ratio >135%) (ROC area under curve = 0.61, 0.43, 0.493, resp.) revealed the better performance of 24-hour urinary aldosterone. *Conclusions*. In conclusion, 24-hour urinary aldosterone level performed better to predict the presence of LVH and inappropriate LVMI in patients with PA.

## 1. Introduction

Primary aldosteronism (PA), characterized by an inappropriate production of aldosterone, can be found in 5–13% of patients with hypertension [1, 2]. PA was once thought to be a rare condition. However, new evidence suggests that PA is the most frequent cause of secondary hypertension [3]. Patients with PA have an inappropriate degree of left ventricular concentric remodeling compared with patients with essential hypertension of a similar level of blood pressure [4–6]. Aldosterone not only stimulates cardiomyocyte growth and hypertrophy but also increases the production of extracellular matrix; thus, leads to increased myocardial fibrosis [7]. Left ventricular hypertrophy (LVH) is a compensatory response to the increased afterload. Initially, LVH reduce the increased stress on the myocardium. However, the left ventricular mass (LVM) may increase disproportionately to the hemodynamic load in patients with PA [4]. The increase in aldosterone levels has been demonstrated to be a possible contribution to this inappropriate increase in LV mass [4]. The appropriateness of LVM to loading condition and body size can be estimated by gender, body height, blood pressure, and stroke volume [4, 8]. In patients with essential hypertension, the presence of inappropriate LVM for a given workload is associated with a higher prevalence of low LV systolic contractility and abnormal relaxation, which suggests a higher risk of transition from compensatory LVH into symptomatic heart failure [8]. The long-term effect of inappropriate LVM in patients with PA is still unknown.

Although aldosterone is directly involved in the pathogenesis of LVH in patients with PA, the plasma aldosterone concentration (PAC), plasma renin activity (PRA), and aldosterone-to-renin ratio were only weakly correlated with LVM in these patient groups [4, 9]. This may be partly caused by the circadian fluctuation of plasma level of aldosterone, with a peak in the early morning. The measurement of 24-hour urinary aldosterone has a potential advantage to estimate the integrated daily exposure to aldosterone, and to avoid the large fluctuation in the circulating plasma aldosterone level. In patients with essential hypertension, 24-hour urine aldosterone level has a better correlation with LVM than plasma aldosterone level and in LV mass [10, 11]. The 24-hour urinary aldosterone has been shown to have a weak correlation with the degree of LVM inappropriateness (*r* = 0.19; *P* = 0.05) in patients with PA [4]. However, the comparison of the performance between plasma aldosterone and 24-hour urinary aldosterone was not conducted in this study. There is still no ideal parameter to predict the presence of LVH or inappropriate LVH in patients with PA. We design this study to elucidate which parameter is more associated with impaired LV geometry in patients with PA and can predict the presence of LVH and inappropriate LVMI more accurately. 

## 2. Methods

### 2.1. Patients

This cross-sectional study enrolled 106 patients diagnosed with PA from October 2006 to March 2010. The patients were evaluated and registered in the Taiwan Primary Aldosteronism Investigation (TAIPAI) database. The database was constructed for quality assurance in one medical center (National Taiwan University Hospital, Taipei, Taiwan) and its three branch hospitals in different cities (National Taiwan University Hospital Yun-Lin branch, Yun-Lin, southern Taiwan; Far-Eastern Memorial Hospital, Taipei; and Taoyuan General Hospital, Taoyuan, mid Taiwan) [12, 13]. The patients were divided into two groups by median level of 24-hour urinary aldosterone (group 2 and group3, with lower and higher urinary aldosterone level, resp.). Another 31 patients with essential hypertension (group 1) were enrolled from the outpatient department of our hospital for comparison. The medical history of the subjects, including demography and current medication, was recorded. The diagnosis of essential hypertension was made by exclusion according to standard algorithms, based on clinical history and biochemical investigations of all detectable forms of secondary hypertension. 

The serum biochemistry was measured when these patients were first evaluated at National Taiwan University Hospital. The concentration of aldosterone was measured by radioimmunoassay with commercial kits (Aldosterone Maia Kit, Adaltis Italia S.P.A., Bologna, Italy) [14]. The limit of detection was 10.0 pg/mL at a 90% confidence interval. The normal range of aldosterone is 70–350 pg/mL in the upright position. The plasma renin activity was measured by the generation of angiotensin I in vitro using a commercially available RIA kit (Stillwater, MN, USA). Its normal range was 2.63 ± 1.32 ng/mL/h in the upright position. The intra-assay coefficient of variability for the PRA assay was 1.9 (5.0%) (mean (SD)); interassay coefficient of variability 4.5 (5.2%). The echocardiography and the measurement of serum biochemistry were performed at the first evaluation (within one month period from the outpatient clinic visit). Urine samples were collected and stored at −20C until the measurements were performed. It was acid hydrolysis and then followed the assay procedure as serum. Daily urine amount was recorded and 24 hr urinary aldosterone level was calculated by multiplying urine aldosterone value by the daily urine amount. When interpreting the results of a 24-hour urine collection, we assess the adequacy of collection by quantifying the 24-hour urine creatinine excretion. The 24-hour urine creatinine excretion was between 15 and 20 mg/kg body weight.

### 2.2. Diagnostic Criteria for PA

The diagnosis of PA was established in patients with hypertension if all of the following criteria were met: (a) evidence of autonomous aldosterone production based on a post captopril ARR of greater than 35 ng/dL per ng/mL/h and a plasma aldosterone concentration >10 ng/dL; (b) a positive salt infusing test or TAIPAI score >0.960 (60% probability) [14]; (c) evidence of an adenoma, or diffuse enlargement of the adrenal glands on computerized tomography; and (d) a positive dexamethasone suppression test or lateralization of aldosterone secretion on adrenal vein sampling.

Hypertensive patients with screening ARR of <30 ng/dL per ng/mL/h, plasma aldosterone concentration <25 ng/dL, and a negative salt loading test result were diagnosed with essential hypertension.

### 2.3. Echocardiography

A Hewlett-Packard 5500 ultrasound system with a S3 transducer (1.0–3.0 MHz) was used in this study. Transthoracic echocardiographic images were acquired in the fundamental imaging mode. Two-dimensional, M-mode Doppler and tissue Doppler ultrasonography were performed in each patient. Chamber dimension, wall thickness, and left ventricular ejection fraction (M-mode) were measured according to the guidelines of the American Society of Echocardiography by one experienced cardiologist [15]. Another cardiologist measured the echocardiographic data off-line to confirm the reproducibility. The interobserver and intraobserver variability were 8.0% and 8.7%, respectively.

Measured LVMI was derived with echocardiography according to the formula of Devereux and Reichek LV  mass = 1.04 × [(septal  thickness + LV  end-diastolic  diameter + posterior  wall  thickness)^3^ −(LV  end-diastolic  diameter)^3^] − 13.6 (gm) [16]. The predicted LVMI was estimated by the equation derived previously: Predicted LVM = 55.37 + 6.64 × height (m^2.7^) + 0.64 × stroke work − 18.07 × gender (where gender was coded as male = 1 and female = 2) [7]. Left ventricle volume was calculated with Tericholz's formula; stroke work was calculated as systolic blood pressure (in mmHg) × stroke volume × 0.0144 [4]. The excess LVMI was defined as measured LVMI−predicted LVMI. The presence of inappropriate LVMI was defined as positive if measured LVMI to predicted LVMI ratio was greater than 135% [4]. LVH was defined according to Devereux's criteria: LVMI ≧ 134 g m^−2^ in men and 110 g m^−2^ in women [17].

### 2.4. Statistical Analysis

All of the continuous variables were expressed as mean ± standard deviation (SD). Comparisons for continuous data between the three groups were made by one-way ANOVA. Differences between proportions were assessed by Chi-square test. Pearson's correlation test was used to analyze the correlation between LVMI and continuous and categorical parameters. Data of urine aldosterone concentration, PAC, PRA, and ARR were log-transformed before the correlation study due to the nonnormality which was determined by the Kolmogorov-Smirnov test. Before further analysis, the log-transformed data was tested again to assure the normality of distribution. A stepwise multivariate linear regression analysis was used to evaluate the relationship between LVMI and different clinical or laboratory variables. The performances of 24-hour urinary aldosterone, plasma aldosterone level, and plasma aldosterone-to-renin activity ratio (ARR) to predict the presence of LVH and inappropriate LVMI were determined by the receiver-operator characteristic (ROC) curve analysis. A two-tailed *P* value below 0.05 was regarded as significant. The statistical analyses were performed with SPSS version 16.0 (SPSS, Chicago, IL, USA).

## 3. Results

Thirty-one patients with essential hypertension were enrolled as Group 1. A total of 106 patients with PA were further divided into two groups by the level of aldosterone in the 24-hour urine sample (Group 2, PA patients with 24-hour urinary aldosterone level below median; Group 3, PA patients with 24-hour urinary aldosterone level above median). The baseline clinical characteristics are shown in Table 1. The median level of urinary aldosterone in patients with PA was 9.25 *μ*g/day. Patients with PA had higher serum aldosterone concentration, lower plasma renin activity, lower serum potassium level, and higher body mass index. There was a trend for higher diastolic blood pressure in patients with PA compared with those in the essential hypertension group. Concerning medication usage, a higher percentage of patients with PA received ACEI or ARB, alpha blocker, beta-blocker, and spironolactone, compared with the essential hypertension group. There were 23 patients (51%) and 25 patients (50%) receiving aldosterone treatment in group 2 and group 3. There were 30 patients (58%) versus of 46 patients (87%) with aldosterone-producing adenoma in group 2 and group 3, respectively (*P* < 0.001).

Patients with PA had thicker LV wall thickness, larger LV mass, and a lower LV ejection fraction (Table 2). The E and A wave in these 3 groups were comparable. The E′ wave by tissue Doppler from the septum was also lower in patients with PA. The number of patients with LVH and inappropriate LVMI was higher in Groups 2 and 3 compared with Group 1. 

In the univariate analysis, LVMI was significantly associated with 24-hour urinary aldosterone level, body mass index, body weight, log-transformed PAC, log-transformed PRA, log-transformed PAC-to-PRA ratio (ARR), systolic and diastolic blood pressure, gender, serum potassium level, the use of alpha-blocker, and the presence of adenoma (Table 3). The correlations of 24-hour urinary aldosterone level, PAC, or ARR with LV structure parameters are shown in Supplementary Table  1 (available online at http://dx.doi.org/10.1155/2013/294594) (group 1, 2, and 3). Compared with PAC, PRA, or ARR, the 24-hour urinary aldosterone level has a better correlation with LV structure parameters. The correlations of 24-hour urinary aldosterone level with LVM, LVMI, measured-to-predicted LVMI ratio, excess LVMI, and ventricular wall thickness were significant in all of the patient groups.

In multivariate analysis, 24-hour urinary aldosterone level, serum potassium level, diastolic blood pressure, and gender were independent factors to predict LVMI in the groups 1, 2, and 3 (Supplementary Table  2). The 24-hour urinary aldosterone level was also a significant predictor for the excess LVMI in whole study population (Supplementary Table  2).

In patients with PA, the univariate analysis revealed that log-transformed urine aldosterone level (*P* = 0.005), serum potassium level (*P* = 0.006), diastolic blood pressure, the presence of adenoma, and alpha blocker were significantly associated with LVMI (Table 4). In the multivariate analysis of LVMI in patients with PA, only log-transformed urine aldosterone level (*P* = 0.025) and serum potassium level (*P* = 0.037) were independent factors for LVMI (Table 5). In the multivariate analysis using excess LVMI as the dependent variable, only log-transformed urine aldosterone level was an independent factor in groups 2 and 3 (Table 5). 

**Figure 1:**
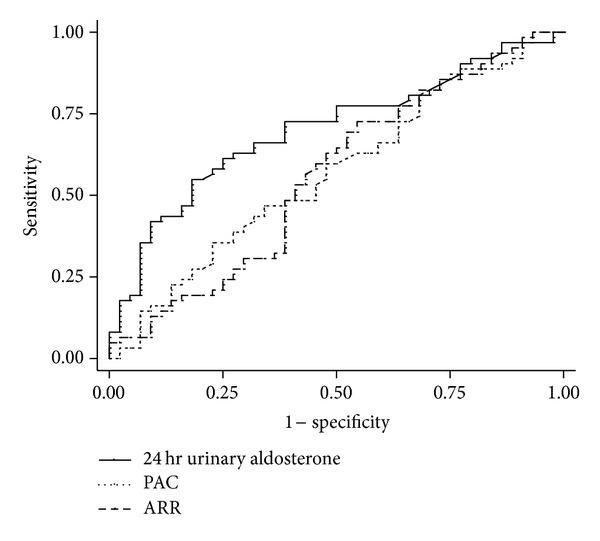
ROC curve: to predict the presence of LVH in patients with primary aldosteronism: the ROC AUC of log-transformed 24 hr urinary aldosterone is larger than that of PAC or ARR. (ROC AUC for log-transformed urine aldosterone: 0.701 (95% CI 0.601–0.801), for PAC: 0.568^†^ (95% CI 0.456–0.68), for plasma aldosterone to renin activity ratio: 0.56^¶^ (0.445–0.675), ^†^ *P* < 0.01  ^¶^ *P* < 0.05, compared with log-transformed urine aldosterone).

**Figure 2:**
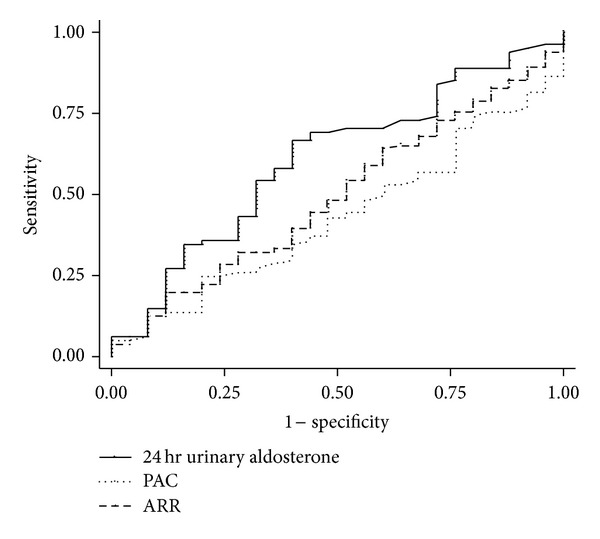
ROC curve: to predict the presence of inappropriate LVMI in patients with primary aldosteronism: the ROC AUC of log-transformed 24 hr urinary aldosterone is larger than that of PAC or ARR. (ROC AUC for log-transformed 24 hr urine aldosterone: 0.61 (95% CI 0.48–0.734); for PAC: 0.43 (95% CI 0.31–0.551); for plasma aldosterone to renin activity ratio: 0.493 (95% CI 0.36–0.61), ^†^ *P* < 0.01 compared with log-transformed urine aldosterone) The presence of inappropriate LVMI is defined as positive if measured to predicted LVMI ratio >135%.

The performance to predict the presence of LVH and inappropriate LVMI is shown in Figures 1 and 2. Comparing the accuracy to predict the presence of LVH, the receiver operating characteristic (ROC) area under curve (AUC) of urine aldosterone was larger than that of PAC, especially in the PA patients (in groups 2 and 3: 24-hour urinary aldosterone AUC = 0.701 (95% CI 0.601–0.801) versus PAC AUC = 0.568 (95% 24 CI 0.456–0.68), *P* = 0.05). Comparing the accuracy to predict the presence of inappropriate LVMI (measured to predicted LVMI >135%), the ROC AUC of 24-hour urinary aldosterone was also larger than that of PAC or ARR (in groups 2 and 3: 24-hour urinary aldosterone AUC = 0.61 (95% CI 0.48–0.734) versus PAC AUC = 0.43 (95% CI 0.31–0.551), *P* = 0.028). The ROC AUC of 24-hour urinary aldosterone is not significantly higher than that of PAC or ARR in patients with essential hypertension (group 1).

## 4. Discussion

The major findings of our study were (1) the 24-hour urinary aldosterone level was significantly correlated with LV structure parameters in patients with PA; (2) the 24-hour urinary aldosterone level was an independent factor for the LVMI and excess LVMI (not explained by gender, body size, and blood pressure) in patients with PA; and (3) the accuracy of 24-hour urinary aldosterone to the presence of inappropriate LVMI was better than that of baseline PAC or ARR in patients with PA.

Although aldosterone has long been connected to excessive LVH in patients with PA, the correlation of baseline plasma aldosterone level and LVMI is not strong in the literature [4, 18]. In our study, baseline PAC was not significantly related to LVMI and only marginally correlated to the excess LVMI. In contrast, urine aldosterone level was significantly correlated to both LVMI and excess LVMI. In the multivariate analysis, urine aldosterone was repeatedly shown to be an independent factor for LVMI and excess LVMI. In short, urine aldosterone level had higher accuracy to diagnose LVH.

Since plasma aldosterone level is influenced by many factors, such as sodium intake, medication [19], or circadian rhythm [20], a single time point plasma level might not have a good correlation with the chronic exposure of myocardium to aldosterone over a long period. The 24-hour urinary aldosterone level may reflect the exposure of aldosterone more accurately than baseline PAC. Our observation is in line with previous research on patients with essential hypertension; while the 24-hour urinary aldosterone level was significantly correlated with LV mass, the PAC was not significantly correlated with LV structure in patients with essential hypertension [10]. Although 24-hour urinary aldosterone has been found to be correlated with inappropriate LVMI in the study of Muiesan et al., the correlations between 24-hour urinary aldosterone with LVMI and other LV structural parameters were not available in this study [4]. The relative performance between plasma aldosterone and 24-hour urinary aldosterone was shown in our study. Our data clearly revealed that 24-hour urinary aldosterone level had a better performance to identify LV structural change in patients with PA. There are some possible explanations for the differences in the results. First, the prevalence of LVH may be slightly higher in our population (LVH *n* = 71, 52%). As the duration from disease onset to the performance of the previous study was unknown, it is possible that the correlation may be stronger in a population with a longer established hypertrophy. Second, the number of patients with adenoma was also higher in our population (adenoma, *n* = 76, 71% of groups 2 and 3). We cannot rule out the correlations between patients with adenoma and patients with hyperplasia are different.

Patients with PA are associated with a high cardiovascular complication rate disproportional to their blood pressures [21]. The LV mass of patients with PA has also been shown to be out of proportion to the extent that can be induced by hemodynamic load and body size [3, 4]. These two pieces of evidence affirm the importance of looking at the inappropriateness of LV mass in patients with PA and the translation of these changes into excessive cardiovascular events. In patients with essential hypertension, the inappropriateness of LV mass has been shown to predict cardiovascular risk independently of age and blood pressure [8, 22]. According to our observation, the presence of inappropriate LVMI may occur before the presence of LVH. Whereas data concerning the long-term prognostic value of the presence of inappropriate LVMI in patients with PA is still pending, based on our observation, it would be reasonable to monitor 24-hour urinary aldosterone level for future cardiovascular complications.

Aldosterone has been shown to have a direct hypertrophic effect on cardiomyocytes. In neonatal rat ventricular myocytes, aldosterone caused a 27% increase in protein incorporation and a 29% increase in cardiomyocyte surface area. This was also associated with increased mRNA levels of *α*- and *β*-myosin heavy chain [23]. Furthermore, aldosterone increases interstitial fibrosis in myocardium. In vitro data revealed that aldosterone increases the collagen synthesis in cardiac fibroblast [24]. In our previous report using integrated backscatter on echocardiography, the increased amount of collagen content in patients with PA could be reversed after adrenalectomy [13].

There are several limitations in this study. First, the small patient number in this study prevents us from drawing definite conclusions. A further large-scale study is needed to confirm our results. Second, this is a cross-sectional study without follow-up data. Whether urinary aldosterone can predict long-term cardiovascular events better than other parameters is still unknown. Third, the data after operation are not available in this study. Further study to investigate the urinary aldosterone level and the regression in LVH and inappropriate LVMI after surgery is warranted. Fourth, the aldosterone is secreted mainly as tetrahydroaldosterone and 10% as aldosterone-18-glucuronide and less than 5% aldosterone is secreted in the free aldosterone. Other aldosterone metabolites are unknown or seldom routinely measured. We did not check the tetrahydroaldosterone level in our study, mainly because of the cost. However, our results based on the urinary aldosterone level provide a simple and feasible way to identify LVH and inappropriate LVMI in clinical practice. Fifth, the urinary sodium level was not measured in our study. The relationship between urinary sodium, 24 hr urinary aldosterone level, and LVH could not be determined. The effect of the antihypertensive drugs effect also could not be fully excluded. Finally, although the 24-hour urinary aldosterone level is the only factor that significantly correlated with left ventricular structure parameters in patients with essential hypertension (Supplementary Table  1), the performance to identify the presence of LVH or inappropriate LVMI was not significantly higher than that for PAC or ARR in this patient group. This statistical nonsignificance may be due to the small number of patients in this group.

## 5. Conclusion

In conclusion, our results show that 24-hour urinary aldosterone is significantly correlated with LV structural parameters including LV wall thickness and LV mass. The inappropriate LV mass, exceeding the amount required to compensate for the loading condition, is also correlated with 24-hour urinary aldosterone. The 24-hour urinary aldosterone is a better indicator for the presence of inappropriate LVMI in patients with PA compared with PAC or ARR.

## Supplementary Material

Supplementary Table 1: shows the correlations of 24-hour urinary aldosterone level, plasma aldosterone concentration (PAC), plasma renin activity(PRA) or aldosterone-to-renin ratio (ARR) with left ventricular structure parameters (group 1, 2 and 3). Compared with PAC, PRA or ARR, the 24-hour urinary aldosterone level has a better correlation with LV structure parameters. The correlation of 24-hour urinary aldosterone level with left ventricular mass index (LVMI), measured-to-predicted LVMI ratio, excess LVMI and ventricular wall thickness were significant in all of the patient groups.
Supplementary Table 2: shows the predictors for left ventricular mass index and excess left ventricular mass index: 24-hour urinary aldosterone level is an independent predictor in all patient groups.

## Figures and Tables

**Table 1:** Clinical data of patients.

Group	1 (*n* = 31)	2 (*n* = 53)	3 (*n* = 53)	*P*
Age, years, mean (SD)	50.5 (15.6)	50.9 (11.4)	46.9 (12.6)^§^	0.209
Men, *n* (%)	11 (35.5)	26 (47.3)	24 (43.6)	0.569
History of hypertension, years, mean (SD)	5.2 (5.0)	8.0 (7.6)	6.6 (7.8)	0.251
Body weight, kg, mean (SD)	62.7 (12.0)	67.2 (14.0)	66.9 (13.0)	0.293
Body height, cm, mean (SD)	162.2 (9.7)	161.0 (8.3)	161 (8.8)	0.744
Body mass index, kg/m^2^, mean (SD)	23.7 (2.6)	25.9 (4.0)^†^	25.7 (3.5)^¶^	0.019
Blood urea nitrogen, mg/dL, mean (SD)	13.6 (3.1)	14.0 (4.2)	12.1 (3.8)^§^	0.052
Creatinine, mg/dL, mean (SD)	0.94 (0.17)	0.97 (0.23)	0.91 (0.24)	0.472
SBP, mmHg, mean (SD)	146 (17)	152 (22)	153 (22)	0.293
DBP, mmHg, mean (SD)	87 (11)	94 (13)^†^	93 (14)	0.056
Serum potassium, mEq/L, mean (SD)	4.21 (0.33)	3.53 (0.67)^†^	3.47 (0.78)^¶^	<0.001
PAC, ng/dL, mean (SD)	24.3 (11.1)	48.9 (39.5)^†^	57.0 (37.6)^¶^	<0.001
PRA, ng/mL/h, mean (SD)	7.5 (8.8)	0.7 (0.9)^†^	0.8 (1.6)	<0.001
PAC*, mean (SD)	1.34 (0.18)	1.59 (0.26)^†^	1.67 (0.27)^¶^	<0.001
PRA*, mean (SD)	0.53 (0.6)	−0.52 (0.7)^†^	−0.63 (0.8)^¶^	<0.001
24-hour urinary aldosterone level, mean (SD)	6.5 (2.4)	6.1 (1.4)	23.7 (12.4)^¶§^	<0.001
24-hour urinary aldosterone level*, mean (SD)	0.79 (0.14)	0.77 (0.11)	1.31 (0.22)^¶§^	<0.001
Number of hypertension medication, mean (SD)	1.7 (0.9)	2.0 (1.0)	1.8 (1.0)	0.8
Hypertension medication:				
CCB, *n* (%)	17 (55)	30 (57)	29 (55)	0.598
ACEI or ARB, *n* (%)	19 (62)	13 (25)^†^	15 (28)^¶^	0.004
Thiazide, *n* (%)	3 (10)	2 (3.7)	5 (9.4)	0.547
*α*-blocker, *n* (%)	3 (10)	14 (26.4)^†^	22 (41.5)^¶^	0.006
*β*-blocker, *n* (%)	9 (29)	28 (52.8)^†^	19 (35.8)^§^	0.01
Spironolactone, *n* (%)	0 (0)	23 (43)^†^	25 (47.2)^¶^	<0.001
Adenoma, *n* (%)		30 (58)	46 (87)^§^	<0.001

*log-transformed.

**SBP: systolic blood pressure; DBP: diastolic blood pressure; PAC: plasma aldosterone concentration; PRA: plasma renin activity; CCB: calcium channel blocker; ACEI: Angiotensin converting enzyme inhibitor; ARB: angiotensin receptor blocker.

^†^Group 2 versus group 1, *P* < 0.05.

^¶^Group 3 versus group 1, *P* < 0.05.

^§^Group 3 versus group 2, *P* < 0.05.

**Table 2:** Echocardiographic data.

Group	1	2	3	*P*
IVST, mean (SD)	10.3 (2.2)	11.4 (2.0)^†^	12.4 (2.2)^¶§^	<0.001
LVPWT, mean (SD)	9.4 (1.3)	10.8 (1.7)^†^	11.6 (1.7)^¶§^	<0.001
LVEDD, mean (SD)	45.5 (4.8)	45.6 (5.2)	46.4 (5.2)	0.649
LVESD, mean (SD)	26.0 (5.1)	28.4 (4.7)^†^	27.9 (4.7)	0.098
LVM, mean (SD)	180.1 (55.3)	220.5 (85.1)	249.7 (76.0)^¶^	<0.001
LVMI, g/m^2^, mean (SD)	106.3 (27.1)	127.0 (42.4)^†^	144.3 (38.0)^¶§^	<0.001
LVEF, mean (SD)	73.0 (9.1)	67.1 (7.5)^†^	69.6 (7.6)	0.008
E, mean (SD)	74.6 (14.9)	75.0 (16.4)	72.4 (17.2)	0.695
A, mean (SD)	70.2 (18.1)	74.2 (14.0)	73.8 (19.6)	0.550
E/A, mean (SD)	1.14 (0.34)	1.04 (0.28)	1.03 (0.33)	0.293
E′ (septal), mean (SD)	8.5 (1.7)	7.5 (2.5)^†^	7.1 (2.1)^¶^	0.018
A′ (septal), mean (SD)	10.5 (1.8)	11.0 (2.6)	10.5 (2.1)	0.494
Stroke volume index (mL/m^2^), mean (SD)	41.8 (9.7)	38.1 (9.3)	40.9 (9.8)	0.164
LVH, *n* (%)	9 (29)	23 (43.4)	39 (73.5)^¶§^	<0.001
iLVMI, *n* (%)	11 (35)	37 (70)^†^	44 (83)^¶§^	<0.001

IVST: interventricular septal thickness; LVPWT: left ventricular posterior wall thickness; LVEDD: left ventricular end-diastolic diameter; LVESD: left ventricular end-systolic diameter; LVM: left ventricular mass; LVMI: left ventricular mass index; LVEF: left ventricular ejection fraction; LVH: left ventricular hypertrophy, defined as LVMI ≥ 134 g m^−2^ in men and 110 g m^−2^ in women; iLVMI: inappropriate LVMI, defined as positive if measured LVMI to predicted LVMI ratio was greater than 135%.

^†^Group 2 versus group 1, *P* < 0.05.

^¶^Group 3 versus group 1, *P* < 0.05.

^§^Group 3 versus group 2, *P* < 0.05.

**Table 3:** Factors associated with LVMI in groups 1, 2, and 3 patients.

Factor	Pearson's correlation	*P*
Urine aldosterone	0.359	<0.001
Urine aldosterone to plasma renin activity ratio	0.287	0.001
Plasma aldosterone to renin ratio	0.223	0.009
Plasma aldosterone concentration	0.143	0.096
Plasma renin activity	−0.197	0.022
Body mass index	0.026	0.016
Body weight	0.202	0.018
Systolic blood pressure	0.233	0.01
Diastolic blood pressure	0.295	0.001
Age	−0.048	0.579
Sex	0.196	0.022
Adenoma	0.264	0.007
Serum potassium	−0.354	<0.001
Alpha blocker	0.208	0.02

**Table 4:** The correlation of urine aldosterone level and LV wall thickness, dimension, and LA size in patients with PA (groups 2 and 3).

	LVMI	Excess LVMI^†^	LV mass	IVS	PW	LVEDD	LVESD	RWT	LA
Urine aldosterone	0.281*	0.265*	0.286*	0.300*	0.298*	0.149	0.037	0.150	0.170
PAC	0.020	0.057	0.019	0.064	0.094	−0.069	−0.061	−0.031	0.029
PRA	−0.046	−0.077	0.012	−0.043	−0.036	0.099	0.078	−0.179	−0.041
ARR	0.050	0.090	−0.005	0.061	0.064	−0.115	−0.093	0.157	0.048

IVS: interventricular septum; PW: posterior wall; LVEDD: left ventricular end-diastolic diameter; LVESD: left ventricular end-systolic diameter; RWT: relative wall thickness = 2 × PW/LVEDD; LA: left atrium.; PAC: plasma aldosterone concentration; PRA: plasma renin activity; ARR: plasma PAC-to-PRA ratio.

*P < 0.05.

^†^Excess LVMI = measured LVMI − predicted LVMI.

**Table 5:** Predictors for LVMI or inappropriate LVMI in patients with PA (groups 2 and 3).

Independent variables	*B* (95% CI)	*P*
(A) LVMI as the dependent factor		
Urine aldosterone	29.9 (8.6–51.3)	0.006
Serum potassium	−13.4 (−23–−3.7)	0.007
Diastolic BP	0.629 (0.147–1.112)	0.011
Sex	13.7 (1.15–26.33)	0.033
(B) Excess LVMI^¶^ as the dependent factor		
Urine aldosterone	29.9 (10.6–49.2)	0.003
Serum potassium	−13.6 (−22–−4.8)	0.003

^¶^Excess LVMI = measured LVMI − predicted LVMI.
