# Supplementary material for: Twenty-Four-Hour Urinary Aldosterone Predicts Inappropriate Left Ventricular Mass Index in Patients with Primary Aldosteronism
Source: ScientificWorldJournal. 2013 Dec 29;2013:294594. doi: 10.1155/2013/294594 (PMC3891226; doi:10.1155/2013/294594)
Supplement: Supplementary file 1 — Supplementary Table 1: shows the correlations of 24-hour urinary aldosterone level, plasma aldosterone concentration (PAC), plasma renin activity(PRA) or aldosterone-to-renin ratio (ARR) with left ventricular structure parameters (group 1, 2 and 3). Compared with PAC, PRA or ARR, the 24-hour urinary aldosterone level has a better correlation with LV structure parameters. The correlation of 24-hour urinary aldosterone level with left ventricular mass index (LVMI), measured-to-predicted LVMI ratio, excess LVMI and ventricular wall thickness were significant in all of the patient groups. Supplementary Table 2: shows the predictors for left ventricular mass index and excess left ventricular mass index: 24-hour urinary aldosterone level is an independent predictor in all patient groups. [file 294594.f1.pdf]

Supplementary Table 1: The correlation of urine aldosterone level and LV wall thickness, dimension and LA size in Group 1 only and in Group 2 and 3

***Group 1	LVMI	Measure to predicted LVMI ratio¶	excess LVMI	LV mass	IVS	PW	LVEDD	LVESD	RWT	LA
Urine Aldosterone*	0.428*	0.345*	0.386*	0.477*	0.480*	0.392*	0.176	0.119	-0.289	0.277
PAC*	-0.014	-0.037	-0.042	0.048	-0.032	0.070	0.045	0.148	-0.027	0.190
PRA*	-0.087	-0.083	-0.073	-0.002	-0.073	0.049	0.026	0.079	-0.274	-0.348
ARR*	0.011	-0.026	-0.048	-0.008	-0.126	-0.282	0.231	0.367*	-0.476	0.201

Group 1, 2, 3	LVMI	Measure to predicted LVMI ratio¶	excess LVMI	LV mass	IVS	PW	LVEDD	LVESD	RWT	LA
Urine Aldosterone	0.359*	0.350*	0.369*	0.363*	0.373*	0.395*	0.152	0.100	0.165	0.325*
PAC	0.143	0.224*	0.207*	0.148	0.170*	0.259*	-0.023	0.061	0.008	0.264*
PRA	-0.197*	-0.223*	-0.223*	-0.110	-0.162	-0.185*	0.054	-0.006	-0.231	-0.298*
ARR	0.223*	0.293*	0.286*	0.172*	0.197*	0.265*	-0.013	0.110	0.196*	0.325*

Supplementary Table 2: predictors for LVMI or inappropriate LVMI in Group 1 only or Group 2 and 3

†Group 1		
A. LVMI as the dependent factor		
Independent variables	B (95% CI)	P
Urine aldosterone	81.9(26.6-137.2)	0.005
K	-37.6(-61.8- -13.4)	0.004
SBP	0.569(0.142-0.997)	0.011
B. excess LVMI¶ as the dependent factor		
Independent variables	B (95% CI)	P
Urine aldosterone	68.32(12.4-124.2)	0.019
K	-37.9(-63.2 - -12.6)	0.005
†Groups 2, 3		
A. LVMI as the dependent factor		
Independent variables	B (95% CI)	P
Urine aldosterone	29.3(3.2-55.4)	0.025
K	-12.78 (-24.8—0.7)	0.037
B. excess LVMI¶ as the dependent factor		
Independent variables	B (95% CI)	P
Urine aldosterone*	29.2(7.2-51.1)	0.01

¶excess LVMI = measured LVMI – predicted LVMI
